# Xbox Kinect Sports vs. Nintendo Switch Sports and Their Effects on Body Composition and Physical Performance in Older Females: A Randomized Controlled Trial

**DOI:** 10.3390/jcm13174987

**Published:** 2024-08-23

**Authors:** Jordan Hernandez-Martinez, Izham Cid-Calfucura, Tomás Herrera-Valenzuela, Nicole Fritz-Silva, Julio B. Mello, Joaquin Perez-Carcamo, Edgard Vásquez-Carrasco, Eugenio Merellano-Navarro, Braulio Henrique Magnani Branco, Pablo Valdés-Badilla

**Affiliations:** 1 Department of Physical Activity Sciences, Universidad de Los Lagos, Osorno 5290000, Chile; jordan.hernandez@ulagos.cl; 2 G-IDyAF Research Group, Department of Physical Activity Sciences, Universidad de Los Lagos, Osorno 5290000, Chile; joaquinalejandro.perez@alumnos.ulagos.cl; 3 Programa de Investigación en Deporte, Sociedad y Buen Vivir, Universidad de los Lagos, Osorno 5290000, Chile; 4 Department of Physical Activity, Sports and Health Sciences, Faculty of Medical Sciences, Universidad de Santiago de Chile (USACH), Santiago 8370003, Chile; izham.cid@usach.cl (I.C.-C.); tomas.herrera@usach.cl (T.H.-V.); 5 Department of Health, Universidad de Los Lagos, Osorno 5290000, Chile; nicole.fritz@ulagos.cl; 6 eFiDac Research Group, School of Physical Education, Pontificia Universidad Católica de Valparaíso, Valparaíso 2340025, Chile; julio.mello@pucv.cl; 7 Occupational Therapy School, Faculty of Psychology, University de Talca, Talca 3460000, Chile; edgar.vasquez@utalca.cl; 8 Department of Physical Activity Sciences, Faculty of Education Sciences, Universidad Católica del Maule, Talca 3530000, Chile; emerellano@ucm.cl; 9 Graduate Program in Health Promotion, Cesumar University (UniCesumar), Maringá 87050-900, Brazil; braulio.branco@unicesumar.edu.br; 10 Sports Coach Career, School of Education, Universidad Viña del Mar, Viña del Mar 2580022, Chile

**Keywords:** virtual reality, exergaming, aging, physical functional performance, exercise

## Abstract

**Background/Objectives**: This study aimed to compare the effects of Xbox Kinect Sports (XKS) regarding Nintendo Switch Sports (NSS) and an inactive control group (CG) on body composition (body fat percentage, BFP; and fat-free mass) and physical performance (maximal isometric handgrip strength, MIHS; 30-s chair stand, 30-S; timed up-and-go, TUG; sit-and-reach; and 2-min step) in physically inactive older females. **Methods:** A randomized controlled trial study was conducted with three parallel groups: XKS (n = 13), NSS (n = 14), and CG (n = 16) considering three weekly 60-min sessions for 12 weeks with pre- and post-assessments. **Results:** A two-factor mixed analysis of variance (ANOVA) model with repeated measures was performed to measure the time × group effect. Multiple comparisons revealed significant differences in BFP (F_(2,18)_ = 6.12; *p* = 0.005; ηp^2^ = 0.226, large effect), 30-S (F_(2,18)_ = 20.7; *p* = 0.000; ηp^2^ = 0.496, large effect), TUG (F_(2,18)_ = 10.0; *p* = 0.000; ηp^2^ = 0.323, large effect), sit-and-reach (F_(2,18)_ = 37.3; *p* = 0.000; ηp^2^ = 0.640, large effect), and 2-min step (F_(2,18)_ = 9.85; *p* = 0.000; ηp^2^ = 0.319, large effect) in favor of XKS regarding NSS and CG. The intragroup results only present in XKS a significant decrease in BFP (*p* = 0.02; d = 0.98) and significant improvements in the 30-S (*p* = 0.000; d = 1.88), TUG (*p* < 0.01; d = 2.00), sit-and-reach (*p* = 0.003; d = 2.58), and 2-min step (*p* = 0.004; d = 1.05). **Conclusions:** training using XKS significantly decreases BFP and improves 30-S, TUG, sit-and-reach, and 2-min step in physically inactive older females.

## 1. Introduction

Low physical activity levels harm the health status of older people, with 31% of this aging population being physically inactive worldwide [1]. These findings showed that body fat percentage increased while fat-free mass fell between 13% and 16% [2,3], and there were changes in physical performance [3,4], such as in muscle strength, postural balance, and gait speed [2,3]. However, maintaining an active and healthy lifestyle as one ages by regularly engaging in moderate (≥150 to 300 min/week) or vigorous (≥75 to 150 min/week) physical activity leads to a decrease in body fat percentage and an increase in fat-free mass [5,6], as well as improved physical performance, in older people [6].

It is, therefore, essential to carry out physical activity interventions in older people [7]. One of the most successful interventions in older people has been through non-immersive virtual reality such as active exergames, as it is a novel and entertaining alternative, with high enjoyment and adherence to the activity in a reduced space [8,9,10], where motivation by performing repetitive tasks offering continuous feedback leads to an adaptive environment for older people [9,11]. These active exergame interventions have reported significant improvements in body composition and physical performance in older people [11,12]. In a study conducted by Biesek, Vojciechowski, Filho, Menezes Ferreira, Borba, Rabito, and Gomes [12] in pre-frail older people, significant improvements in fat-free mass (*p* = 0.02) and maximal isometric handgrip strength (MIHS, *p* = 0.004) were reported compared to an inactive control group. In an overview conducted by Hernandez-Martinez, Ramos-Espinoza, Muñoz-Vásquez, Guzman-Muñoz, Herrera-Valenzuela, Branco, Castillo-Cerda, and Valdés-Badilla [11] in older people, significant improvements were reported in the Berg balance scale (*p* = 0.02), timed up-and-go (TUG, *p* < 0.0001), and 30-s chair stand (*p* = 0.0008) in comparison with active/inactive control groups. A study by Queiroz et al. [13] compared active exergames vs. aerobic exercises in older people, showing significant improvements in both groups in 30-s chair stand (*p* < 0.05) and TUG (*p* < 0.05). On the contrary, a study conducted by Padala et al. [14] in apparently healthy older people reported significant improvements in the Berg balance scale (*p* < 0.001) in favor of active exergames compared to an active control group that performed cognitive exercises. In addition, interventions using active exergames have shown a greater enjoyment of the exercise than conventional physical activity [15].

The virtual reality consoles most commonly used in studies with older people are Nintendo Wii^®^, with the Wii Sports, Wii Balance, and Wii Fit games [16,17,18]; Xbox Kinect 360^®^, from Microsoft, with the games Kinect Sports, Adventure, and Your Shape [16,18]; and Nintendo Switch^®^, with the game Switch Sports [19]. Several studies have analyzed the effect of exergames (Nintendo Wii, Xbox Kinect, and Nintendo Switch) on functional capacity compared to active/inactive control groups [19,20,21]. In a study conducted by Keogh, Power, Wooller, Lucas, and Whatman [21] in older residential people, they reported significant improvements in bicep curl muscle endurance (*p* < 0.05), physical activity levels (*p* < 0.05), and the psychological dimension of health-related quality of life (*p* < 0.05) in favor of Nintendo Wii Sports compared to an inactive control group. Another study conducted by Hernandez Martínez, Ramirez Campillo, Álvarez, Valdés Badilla, Moran, and Izquierdo [20] in apparently healthy physically inactive older females’ significant improvements in TUG (*p* < 0.01), walking speed (*p* < 0.01), and 5-repetition sit-to-stand test (*p* < 0.01) were reported in favor of Xbox Kinect Sport (XKS) compared to an inactive control group. This was similar to that reported by Zegarra-Ramos, García-Bravo, Huertas-Hoyas, Fernández-Gómez, Rodríguez-Pérez, Pérez-Corrales, and García-Bravo [19] in apparently healthy older people showing significant improvements in functionality of the activities of daily living (*p* = 0.005), physical health (*p* = 0.006), general health perception (*p* = 0.006), and health-related quality of life (*p* = 0.006) in favor of Nintendo Switch Sports (NSS) and conventional occupational therapy.

There is evidence of the benefits of these consoles [19,20,21]. As reported by Wang [22], these can vary in the space in which the game is developed (dimensions) and the place where the sensor is located, for example, XKS in a camera in front of the player and NSS in a control that goes in the hand and/or on the leg of the player as the biomechanics and scenarios to develop the games; this may vary the response, as well as the effects, of the intervention [22]. As far as our knowledge has found, no studies have been conducted comparing similar sports games on different consoles that offer different combinations of both oculo-manual and oculo-foot coordination movements used by older people. Considering that females worldwide live longer than males and have a higher risk of functional dependence, it is important to implement interventions to improve body composition, physical performance, and quality of life [23]. Therefore, this study aimed to compare the effects of XKS regarding NSS and an inactive control group (CG) on body composition (body fat percentage and fat-free mass) and physical performance (MIHS, 30-s chair stand, TUG, sit-and-reach, and 2-min step test) in physically inactive older females.

## 2. Methods

### 2.1. Study Design

**Figure 1:**
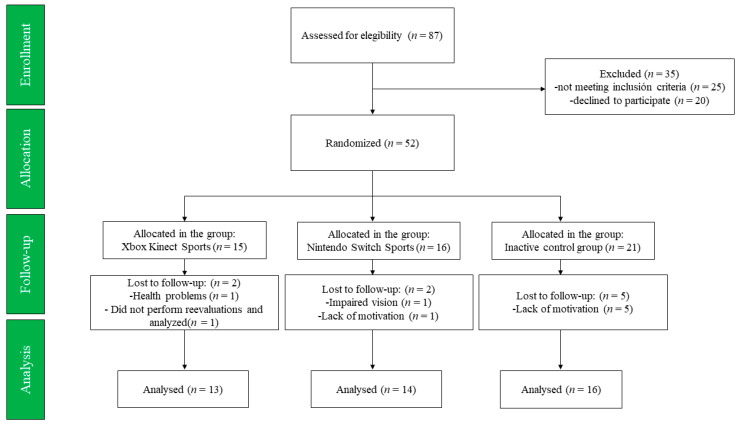
Study flowchart of the enrolment process, allocation, follow-up, and analysis of older females.

A sealed envelope lottery was conducted for randomization with a single-blinded (evaluators) design, three-arm (XKS, NSS, and CG) randomized controlled trial. The randomizer website (https://www.randomizer.org, accessed on 13 August 2024) was used to conduct the randomization process. The CONSORT guidelines method was used [24], i.e., including randomization and sample calculation, definition and complete justification of the trial results, and description of the statistical methods used to compare groups. It was also registered with the US Clinical Trials Registry and Outcomes System (code: NCT06551870; https://clinicaltrials.gov/search?cond=NCT06551870, accessed on 13 August 2024). The interventions were carried out for 3 weekly sessions for 12 weeks (36 sessions) [11]. These sessions were performed thrice a week (Monday, Wednesday, and Friday) for 60 min each. Body fat percentage, fat-free mass, MIHS dominant and non-dominant hands, 30-s chair stand, TUG, sit-and-reach, and 2-min step test were assessed. All measurements were performed in the afternoon, between 15:00 and 17:00 h, and at the same location (a community center in Osorno, Chile). At the same time, the training sessions (XKS and NSS) consisted of playing sports video games for 60 min while the CG continued with their daily life activities. In addition to not experiencing pain prior to the assessments or during the training sessions, the older females did not experience musculoskeletal or cardiorespiratory injuries during the intervention. Six older females dropped out due to lack of motivation, one due to health problems, one due to impaired vision, and one for not completing all the reassessments. The study flowchart is summarized in Figure 1.

### 2.2. Participants

Fifty-two physically inactive older females initially participated in the intervention. Following previous studies [13,20], the minimum difference needed for significant clinical relevance was a mean difference of 0.60 s in TUG, with a standard deviation of 0.20 s. An alpha level of 0.05, a power of 85%, and an anticipated loss of 20% were considered. The sample size calculation indicated that the ideal number of participants per group was 13. We used the Gpower program (version 3.1.9.6, Franz Faul, Universiät Kiel, Kiel, Germany) to calculate the statistical power. The inclusion criteria were the following: (i) older females, between 65 and 75 years of age; (ii) who were not cognitively impaired with a mini-mental State Examination score of ≤21 points [25]; (iii) who did not present any visual difficulty and/or any vestibular alteration that would hinder the performance of the games in front of the screen; (iv) who were independent, defined by having at least a score of 43 points in the Preventive Medicine Examination for the Older People of the Chilean Ministry of Health [26]; (v) who were able to meet the attendance requirement of at least 85% of the sessions scheduled for the intervention; (vi) physically inactive who did not meet the international recommendations for moderate (<150 to 300 min) or vigorous (<75 to 150 min) physical activity [27]; and (vii) physical condition compatible with the practice of physical activity. As for the exclusion criteria, the following were taken into account: (i) being afflicted with some disability; (ii) suffering from a musculoskeletal injury or receiving physical rehabilitation therapy, which prevents them from performing their usual physical activity; and (iii) being temporarily or permanently unable to perform physical activity.

By approving the use of the data for scientific purposes by signing an informed consent form, all participants acknowledged the inclusion criteria for the data’s usage and treatment. The protocol was created by the Declaration of Helsinki and approved by the Scientific Ethics Committee of the Universidad Católica del Maule, Chile (approval number: 29-2022).

### 2.3. Anthropometric Parameters and Sociodemographic Assessments

Bipedal height was measured using a stadiometer (Seca model 220, SECA, Hamburg, Germany; accuracy to 0.1 cm), and body weight was calculated using a mechanical scale (Scale-Tronix, Chicago, IL, USA; accuracy to 0.1 kg) while wearing the barest minimum of clothing [28]. The baseline characteristics of the sample are presented in Table 1.

### 2.4. Body Composition

The body fat percentage and fat-free mass were determined using tetrapolar bioimpedance (InBody 570^®^, Seoul, South Korea) and eight tactile point electrodes. For every measurement, the guidelines provided by the International Society for the Advances in Kinanthropometry (ISAK) were adhered to [28].

### 2.5. Maximal Isometric Handgrip Strength (MIHS)

Previous recommendations state that MIHS was employed [29]. It was found that the best position for the test was seated, with the wrist and forearm in a neutral position, the elbow flexed at a 90-degree angle to the side of the body, the spine aligned, and the shoulders in a neutral position. A portable dynamometer (Jamar^®^, PLUS+, Sammons Preston, Patterson Medical, Warrenville, IL, USA) was used for the test. The dynamometer was positioned in the first position, which promotes contact between the thumb and index finger’s first phalanx, to allow for a firm grip on the device while preserving appropriate closure of the metacarpal phalangeal and interphalangeal joints based on hand size. Every participant tried three times with each hand after a 120-s rest.

### 2.6. 30-s Chair Stand Test

The 30-s chair stand test [30], which measures the number of repetitions made while standing up and sitting on the chair with arms resting on the chest for 30 s, was used to assess the lower limbs’ muscle strength and the ability to perform activities of daily living [31]. The best of the three efforts was achieved after three attempts, with a recovery of 120 s between them.

### 2.7. Timed Up-and-Go Test (TUG)

The TUG test was carried out under previous recommendations [32]. The individual has to exit a chair that supports their arms, walk across a 3-m corridor, turn around, and return to the chair. The TUG is a measure of functional mobility and dynamic balance [33]. They have to run through three trials and quickly record the best one. Using single-beam photocells (Brower Timing System, Draper, UT, USA), two assessors recorded the time; statistical analysis was performed on the best three trials.

### 2.8. Sit-and-Reach Test

Utilizing the sit-and-reach test, flexibility was assessed [34]. Subjects were seated forward in a chair with a fixed back, one leg bent on the floor and the other left straight. The left or right leg may be used to run comfortably; however, the post-test measured the same leg as the pre-test. After correcting the position once again, the case of another bent leg evaluates flexibility by executing two attempts using the higher number to determine the result, according to Lemmink et al. [35].

### 2.9. 2-min Step Test

Cardiorespiratory fitness was measured using the 2-min step test [34]. Participants were instructed to stand straight, and the middle distance between the patella and pelvic bones was marked on a wall with colored tape. Participants stepped in a manner where their knees were raised above the marked spot, and the number of repetitions within 2 min was recorded [34].

### 2.10. Intervention

The procedures from earlier studies [13,20] were followed for implementing the interventions (XKS and NSS). The interventions took place in a community center for older people and were carried out by a physical education teacher with experience and training of 2 years in older people. A week of familiarization with the games was conducted during three 60-min sessions prior to randomization of the groups with the aim of familiarizing individuals with the games and the movements to be performed, as recommended by Rytterström et al. [36], and with the RPE scale of the intensity control. After this, the programs were designed to last 12 weeks (36 sessions), with a 10-min warm-up consisting of low-intensity aerobic activities and joint mobility, a 40-min main part (including XKS and NSS), and a 10-min cooldown using static flexibility exercises.

**Figure 2:**
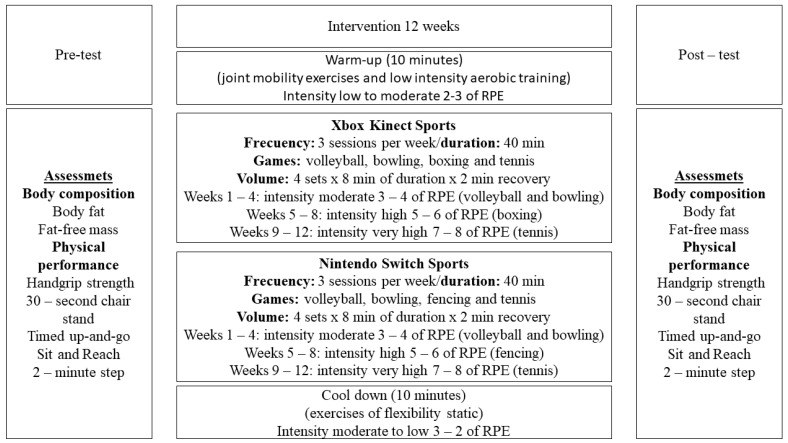
Assessments and regular sessions of the intervention. Note: RPE: rating of perceived exertion.

For XKS, the main part of the training sessions included active exergames of volleyball, bowling, boxing, and table tennis, each lasting 8 min, with 2 min of rest between games. In these active exergames, to run the games, you had to be in front of a sensor in a camera under the television screen in a range of motion 3.5 m wide [37,38]. In NSS, to be able to run the games of these active exergames, you had to have a controller in your hand that did not have a cable because it was connected via Bluetooth to the console, just like, in XKS, you must play in front of the television screen [39]. In NSS, active exergames of volleyball, bowling, fencing, and tennis were carried out in the main part, each lasting 8 min, with 2 min of rest between games. To monitor the intensity of the training, the 10-point rating of perceived exertion (RPE) was used in both groups. The interventions were carried out in the first 4 weeks, with moderate intensities 3 to 4 RPE, progressing from weeks 5 to 8 to high intensities of 5 to 6 RPE and increasing to 7 to 8 RPE in weeks 9 to 12. The complexity of the games was increased by advancing in the levels of the sports games, which led to faster movements with greater demands to achieve progress in the sequences of the games, which allowed to reach increases in intensity. Both XKS and NSS consoles in the execution of their games must perform repetitive movements in different directions that stimulate dynamic balance and cognitive function through constant feedback [11,19,40]. The training sessions were conducted under the physical education teacher’s direct supervision to ensure the exercise protocol’s safety and maintenance. The CG only played board games twice a week for 60 min. Figure 2 presents the summary of the interventions.

### 2.11. Statistical Analysis

Data were analyzed with SPSS 25.0 statistical software (SPSS 25.0 for Windows, SPSS Inc., Chicago, IL, USA). The descriptive statistics included the calculation of the mean and standard deviation. Shapiro–Wilk was applied to determine the data distribution. Subsequently, a two-factor mixed ANOVA model with repeated measures was performed to measure the time × group effect of the body fat percentage, fat-free mass, MIHS dominant and non-dominant hands, 30-s chair stand, TUG, sit-and-reach, and 2-min step test. When the time × group interaction was significant, a Bonferroni multiple comparisons test (post hoc) was performed to establish intragroup (pre vs. post assessments) and intergroup (XKS vs. NSS vs. CG) differences. To determine the effect size of the time × group interaction, the partial eta squared (ηp^2^) was calculated, which was interpreted considering the ηp^2^ values of 0.01, 0.06, and 0.14, which correspond to effect sizes small, moderate, and large, respectively [41]. For multiple comparisons, the effect size was calculated with Cohen’s *d* [42], considering a small (≥0.2), moderate (≥0.5), large (≥0.8), and very large (>1.0) effect. For all analyses, an α value of 0.05 was considered.

The minimal clinically important difference (MCID) of each measure, determined after the intervention, was compared to assess whether intragroup changes were clinically significant. The following MCID values of measures in older people were retrieved from the literature: 3.3 repetitions for 30-s chair stand [43] and one second for the TUG test [44]. For MIHS, there is no clear MCID reported in the literature, with changes of 5.0 to 6.5 kg providing an estimate of meaningful change [45]. To our knowledge, there is no reported MCID for the sit-and-reach and 2-min step tests.

## 3. Results

Table 2 shows the results of the variables before and after the intervention for XKS, NSS, and CG. The two-way mixed ANOVA test revealed a significant time × group interaction for body fat percentage (F_(2,18)_ = 6.12; *p* = 0.005; ηp^2^ = 0.226, large effect), 30-s chair stand (F_(2,18)_ = 20.7; *p* = 0.000; ηp^2^ = 0.496, large effect), TUG (F_(2,18)_ = 10.0; *p* = 0.000; ηp^2^ = 0.323, large effect), sit-and-reach (F_(2,18)_ = 37.3; *p* = 0.000; ηp^2^ = 0.640, large effect), and 2-min step (F_(2,18)_ = 9.85; *p* = 0.000; ηp^2^ = 0.319, large effect). However, in fat-free mass (F_(2,18)_ = 0.30; *p* = 0.74; ηp^2^ = 0.014, small effect), MIHS dominant hand (F_(2,18)_ = 1.03; *p* = 0.36; ηp^2^ = 0.429, large effect), and MIHS non-dominant hand (F_(2,18)_ = 0.27; *p* = 0.76; ηp^2^ = 0.013, small effect), there was no significant interaction.

**Figure 3:**
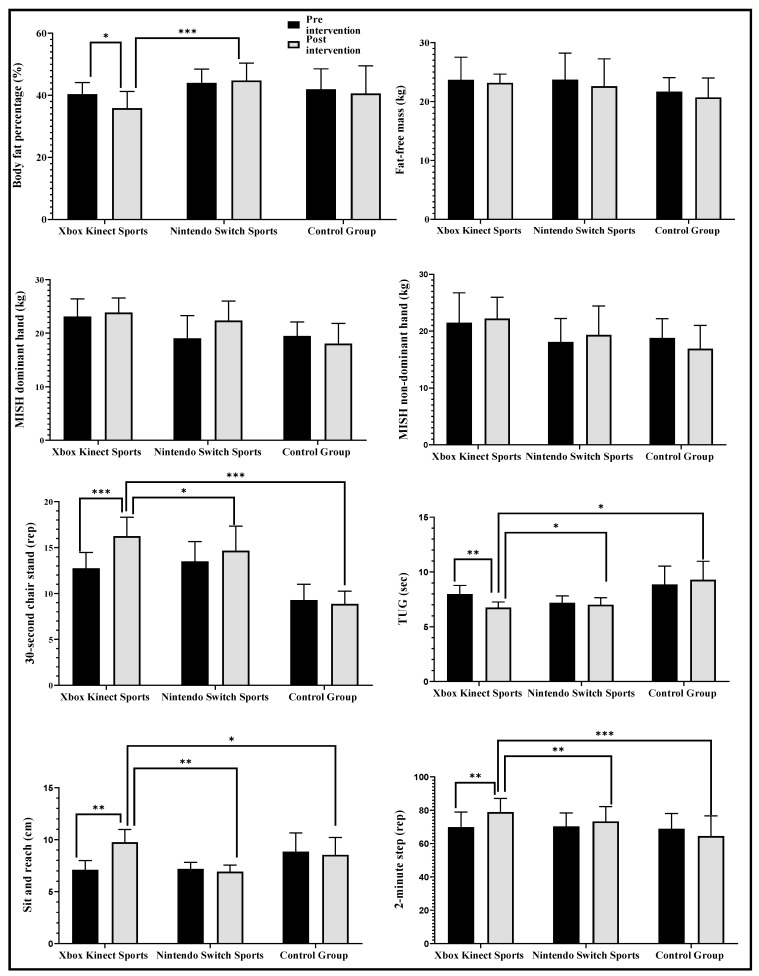
Multiple intragroup and intergroup comparisons of Xbox Kinect Sports vs. Nintendo Switch Sports and the control group on body composition and physical performance. Note: MIHS: maximal isometric handgrip strength. TUG: timed up-and-go. rep: repetitions. * = *p* < 0.05. ** = *p* < 0.01. *** = *p* < 0.001.

The results of the intragroup and intergroup multiple comparisons are shown in Figure 3. Regarding body fat percentage, significant differences were only found in the XKS group before and after the intervention (F_(2,37)_ = 12.1; *p* = 0.02; *d* = 0.98, large effect), with significant differences in favor of the XKS group regarding NSS (*p* = 0.003; *d* = 1.94, very large effect). There were no significant improvements in either group MIHS dominant or non-dominant hands, while, in the 30-s chair stand, a significant increase in XKS (F_(2,37)_ = 0.13; *p* = 0.000; *d* = 1.88, very large effect) was reported in favor of XKS concerning NNS (*p* = 0.01; *d* = 0.68, moderate effect) and regarding CG (*p* = 0.000; *d* = 3.76, very large effect). A significant increase in sit-and-reach was also reported in XKS (F_(2,37)_ = 1.06; *p* = 0.003; *d* = 2.58, very large effect) in favor of XKS concerning NSS (*p* = 0.002; *d* = 2.98, very large effect) and CG (*p* = 0.03; *d* = 0.85, large effect). In the 2-min step test, a significant increase in favor of XKS (F_(2,37)_ = 0.01; *p* = 0.004; *d* = 1.05, very large effect) regarding NNS (*p* = 0.008; *d* = 0.66, moderate effect) and CG (*p* = 0.000; *d* = 1.40, very large effect) was reported, while, in TUG, a significant decrease in XKS (F_(2,37)_ = 3.12; *p* < 0.01; *d* = 2.00, very large effect) was reported concerning NNS (*p* = 0.04; d = 0.45, small effect) and CG (*p* < 0.05; *d* = 0.37, small effect). However, in fat-free mass for MIHS dominant and non-dominant hands, no significant increases were reported in any group.

## 4. Discussion

This study aimed to analyze the effects of XKS regarding NSS and CG on body composition (body fat percentage and fat-free mass) and physical performance (MIHS, 30-s chair stand, TUG, sit-and-reach, and 2-min step test) in physically inactive older females. Significant improvements were reported in favor of XKS over NSS in body fat percentage and physical performance in XKS over NSS and CG in 30-s chair stand, TUG, sit-and-reach, and 2-min step. Significant intragroup improvements were also reported in favor of XKS in the same variables measured above. The clinical significance of these improvements must be considered. For the 30-s chair stand test, which is an indicator of lower limb muscle strength and activities of daily living performance, the average magnitude of improvement of 3.5 sit to stand repetitions was within the range of clinically important values (MCID values from 3.3) [43]; in the TUG test, an indicator of functional mobility and dynamic balance, an improvement of 1.22 s was recorded, a value higher than that considered clinically significant (MCID value of 1 s) [44]. In the short term, mobility and balance may be less responsive to training than muscle strength and daily activities.

No significant increases in fat-free mass were reported for XKS, NSS, and CG. However, in the Wu et al.’s [46] study in older people with dementia, significant increases in fat-free mass were reported in a Nintendo Wii Fit Balance Board intervention lasting 12 weeks with a frequency of 3 sessions per week with a duration of 40 min, as well as an aerobic training intervention; this was different from the findings reported by Rica et al. [47], who, after a 12-week intervention with a frequency of 3 sessions per week with a duration of 60 min based on exercise with Your Shape Fitness Evolved of the Kinect (Xbox 360), did not find increases in fat-free mass in older people. In this regard, variations in the design and methodology of the studies can explain these differences. The sample size, the existence of a CG, and the types of exergames used are important factors in analyzing the available evidence [48]. Second, longer interventions may be necessary to find improvements in fat-free mass [48]. Furthermore, evidence indicates that physical activity plus implementing dietary control with adequate protein intake can improve body composition in older people [49]. Therefore, fat-free mass could be improved with the inclusion of longer active exergame interventions (≥16 weeks) [50] that include dietary control in the participants.

On the other hand, significant decreases were found in favor of XKS regarding NSS in body fat percentage. On the contrary, in a meta-analysis conducted by Deng, Soh, Abdullah, Tan, and Huang [48] in apparently healthy older people, no significant decrease in body fat percentage (*p* = 0.36) was reported in interventions using active exergames (Nintendo Wii Fit and Adventure and Xbox Kinect Adventure) with a duration of 6 to 26 weeks with a frequency of 1 to 3 sessions of 30 to 60 min in duration compared to active/inactive control groups. These were similar results to those reported by Yu et al. [51] in apparently healthy older people where no significant decrease in body fat percentage (*p* = 0.18) was found in a 10-week intervention with a frequency of 3 sessions per week of 50 min using Xbox Kinect Adventure compared to an inactive CG. Similarly, Huang et al. [52], in an intervention using Xbox Kinect with the game Your Shape: Fitness for 12 weeks with a frequency of 3 sessions per week of 30 min, did not report significant decreases in body fat percentage (*p* = 0.08) compared to an inactive CG in apparently healthy older people. Based on our findings, the significant improvements in body fat percentage in the XKS group could be explained by the different gaming devices used. However, they look like identical devices, and Li, Li, Huo, Ma, Wang, and Theng [38] mentioned that it is essential to consider the different connection mechanisms and the form of play. For example, the Wii relies on a motion-sensitive controller to detect three dimensions (3D), as well as detecting a player’s 3D hand posture using a three-axis gyroscopic sensor [38], while Kinect works driver-free and generates motion and voice control using an infrared projector and a camera [38]. In this regard, the Kinect’s lack of a hand controller, unlike the Wii, provides greater freedom of movement and could generate higher levels of physical activity than the Wii; this has been supported by O’Donovan et al. [53], who indicated that playing on Kinect caused greater energy expenditure than playing on Wii among healthy young adults. Finally, Li, Li, Huo, Ma, Wang, and Theng [38] visually reported in their study that Wii participants tended to execute short, sharp movements or movements only of the wrists instead of performing a whole arm and body movement.

However, in MIHS dominant and non-dominant hands, no significant increases were reported in either group, similar to that reported by Hernandez-Martinez, Ramos-Espinoza, Muñoz-Vásquez, Guzman-Muñoz, Herrera-Valenzuela, Branco, Castillo-Cerda, and Valdés-Badilla [11] in an overview of apparently healthy older people where no significant increases in MIHS (*p* = 0.06) were found with interventions of 3 to 20 weeks with a frequency of 2 to 3 sessions per week of 30 to 60 min with active exergames through games (Wii sports, balance and fit, Kinect Sports, Adventure and Your Shape, and Sports Champions Move) in comparison to active/inactive control groups. However, in a study by Liao et al. [54] in apparently healthy older people, significant increases in MIHS dominant and non-dominant hands (*p* < 0.001) were reported by a combined training intervention using XKS with multicomponent training for 12 weeks with a frequency of 3 sessions per week of 60 min compared to only XKS. The interventions with XKS and NSS in our study was demonstrated to not be a sufficient stimulus to improve MIHS in older people; this can be attributed to the execution of upper body movements only with body weight without an added external load [55], which allows increasing the activation and strength of the muscles in the forearm and wrist [56]. In this sense, it is advisable to combine XKS or NSS training with exercises with elastic bands or dumbbells at intensities close to 60% and 80% of the one-repetition maximum for greater neuromuscular activation and muscle hypertrophy [57].

Other results found significant increases in 30-s chair stand in favor of XKS compared to NSS and CG. In a meta-analysis conducted by Taylor et al. [58] in apparently healthy older people, significant increases in 30-s chair stand (*p* = 0.002) were reported in interventions using Nintendo Wii fit and Sports lasting 3 to 20 weeks with a frequency of 2 to 3 sessions for 40 min each compared to active/inactive control groups. Results similar to that reported by Yu, Chiang, Wu, Wu, and Chu [51] in apparently healthy older people showed significant increases in 30-s chair stand (*p* = 0.01) in favor of Xbox Kinect Adventure compared to an inactive control group. As mentioned above, Kinect’s lack of a hand controller, unlike the Wii, provides greater freedom of movement, leading to greater energy expenditure [53]. In this sense, the improvement in favor of the XKS group in the 30-s chair stand can be attributed to the movements with a greater degree of freedom of the lower body performed during volleyball, bowling, boxing, and table tennis games. The actions performed involved participants performing various flexion and extension movements of the lower body while standing and changing positions [48,59,60]; this may have led to an improvement in the neuromuscular function of the lower body, mainly through strengthening of the quadriceps, hamstrings, and glutes [48,60].

Regarding balance, significant improvements in TUG were reported in favor of XKS compared to NSS and CG. Similar results to those reported by Suleiman-Martos et al. [61] in a meta-analysis in apparently healthy older people reported significant improvements in TUG (*p* = 0.002) through interventions with Nintendo Wii fit and Xbox Kinect Adventure lasting 3 to 24 weeks with a frequency of 2 to 3 sessions of 15 to 120 min in duration compared to active/inactive control groups. Similar to that reported by Hernandez Martínez, Ramirez Campillo, Álvarez, Valdés Badilla, Moran, and Izquierdo [20] in apparently healthy physically inactive older females in an intervention using XKS for 8 weeks with a frequency of 3 sessions per week of 30 min in duration reporting significant improvements in TUG (*p* < 0.001) compared to an inactive CG. In the study conducted by Zahedian-Nasab et al. [62] in older people with fall risk, significant improvements in TUG (*p* < 0.001) were reported in an intervention using XKS with a duration of 6 weeks with a frequency of 2 sessions of 60 min in comparison with conventional occupational therapy. Our findings reflect that XKS can be beneficial to improve dynamic balance, given the greater degrees of freedom applied in different directions during video games, causing participants to constantly change their center of pressure [48,63]; in addition, XKS requires the motor control of the participants because the player is only successful in the game if the movements are performed correctly. On the other hand, visual and auditory feedback, along with the increase in difficulty in the games, implies greater participation of the sensorimotor system incorporating all the afferent components, the integration and central processing processes, and the efferent responses, with the aim of maintaining stability of the functional joint during body movements [11,64].

Another result reported was significant improvements in sit-and-reach in favor of XKS compared to NSS and CG. Similar to that reported by Yu, Chiang, Wu, Wu, and Chu [51], healthy older people showed significant improvements in sit-and-reach (*p* = 0.001) in favor of Xbox Kinect Adventure compared to an inactive control group. However, in the study conducted by Liao, Chen, and Wang [54], no significant improvements in sit-and-reach were reported in an intervention with XKS (*p* = 0.59) and XKS combined with multicomponent training (*p* = 0.85) in apparently healthy older people. Similar to what was reported by Wu, Ji, Won, Jo, Kim, and Park [46] in older people with dementia, no significant improvements (*p* > 0.05) were reported in sit-and-reach in an intervention using the Nintendo Wii Balance Board as an intervention through aerobic training. The improvements in the XKS group on the sit-and-reach test may have reflected the execution of continuous movements at different joint angles [65]. Evidence has suggested that flexibility is improved when exercises are performed through a full range of motion and involve agonist and antagonist muscle groups [65]. However, Deng, Soh, Abdullah, Tan, and Huang [48] in a recent systematic review found that active exergames may not be an effective way to increase older people’s flexibility. They argued, in general, that active exergames cannot be used as a substitute for real sports and conventional physical activity.

In cardiorespiratory fitness using the 2-min step test, significant improvements were reported in favor of XKS compared to NSS and CG, similar to what was reported by Wu, Ji, Won, Jo, Kim, and Park [46] in older people with dementia showing significant (*p* < 0.001) improvements in a 2-min step test in an intervention with Nintendo Wii Balance and board compared to aerobic training. Similarly, Wang [66] reported significant improvements in the 2-min step (*p* = 0.01) in a 12-week intervention with a frequency of 3 sessions per week of 90 min using Xbox Kinect with the game Just Dance compared to an inactive CG in apparently healthy older people. Playing games in XKS may generate higher energy expenditure, which could influence vascular function, increasing cardiorespiratory fitness in older people [67]. Recent studies [11,48,67] have reported that the biological mechanisms responsible for the increased cardiorespiratory fitness induced by active exergames could involve adaptations such as improving the heart’s ability to supply oxygen to active muscles, modifying muscle fibers through mechanical and metabolic stress and the promotion of protein synthesis [67,68].

### 4.1. Limitations and Strengths

Our study has some limitations: (i) not controlling the food consumption of the participants; (ii) not measuring physiological variables that may have influenced our results; (iii) not analyzing older males where the results may be different; and (iii) the variability of the difficulty of the games per console could potentially exacerbate the response to intervention due to incorrect movements or overload; therefore, it is relevant in future studies to analyze the individual response to these types of activities by comparing consoles in the same games. Among our strengths are (i) analyzing the effects of two types of active exergames (XKS and NSS), expanding the existing evidence on their effects on the health status of older people, and (ii) comparison with an inactive CG that increases the quality in the comparison on the effects on the analyzed variables.

### 4.2. Practical Applications

In physically inactive older women, XKS training can be a safe and effective method to achieve significant and clinically relevant improvements in both body fat percentage reduction and functional physical performance improvement. These results should be considered when designing better and more appropriate training programs for older people, as XKS training is an affordable and low-cost alternative for community centers and preventive health units working with this population. This type of console can provide greater mobility in the execution of the games compared to NSS and is more economical.

## 5. Conclusions

Training using XKS significantly decreases the body fat percentage and improves the 30-s chair stand, TUG, sit-and-reach, and 2-min step test. Therefore, we recommend the use of active exergames through XKS as an alternative to training physically inactive older females.

## Figures and Tables

**Table 1:** Baseline anthropometric parameters and sociodemographic assessments of physically inactive older females.

Variable	Assessment	XKS (n = 13)	NSS (n = 14)	CG (n = 16)
Age (years)		69.6 (3.16)	73.4 (3.29)	72.1(5.05)
Anthropometric parameters	Bipedal height (cm)	1.55 (0.10)	1.48 (0.09)	1.52 (0.19)
	Body mass (kg)	73.4 (36.4)	74.1 (16.5)	75.5 (6.69)
Academic level	Primary	20%	15%	20%
	Secondary	10%	13%	12%
	Bachelor	4%	3%	3%
	Postgraduate	0%	0%	0%
Civil status	Married	30% *	6%	8%
	Separated	8%	20% *	22% *
	Widowed	1%	4%	1%
	Single	0%	0%	0%

Note: XKS: Xbox Kinect sports. NSS: Nintendo Switch sports. CG: control group. Data are presented as the mean and standard deviation. * = *p* < 0.05.

**Table 2:** Time × group interaction in the analyzed variables of Xbox Kinect Sport, Nintendo Switch Sport, and an inactive control group on body composition and physical performance.

Assessment	Group	Before		After		Time × Group *p*-Value	Time × Group F-Value	ηp^2^	Classification
		Mean	SD	Mean	SD				
Fat-free mass (kg)	XKS	23.7	3.8	23.1	1.4	0.74	0.30	0.014	Small effect
	NSS	23.7	4.4	22.6	4.6				
	CG	21.6	2.3	20.7	3.3				
Body fat percentage (%)	XKS	40.3	3.7	35.8	5.3	0.005	6.12	0.226	Large effect
	NSS	44.0	4.4	44.7	5.5				
	CG	41.1	6.6	40.5	8.8				
MIHS dominant hand (kg)	XKS	23.1	3.27	23.8	2.7	0.36	1.03	0.429	Large effect
	NSS	19.0	4.2	22.3	3.6				
	CG	19.4	2.6	18.0	3.7				
MIHS non- dominant hand (kg)	XKS	21.4	5.2	22.2	3.7	0.76	0.27	0.013	Small effect
	NSS	18.1	4.1	19.3	5.0				
	CG	18.8	3.3	16.9	4.1				
30-s chair stand (rep)	XKS	12.7	1.7	16.2	2.0	0.000	20.7	0.496	Large effect
	NSS	13.5	2.1	14.6	2.6				
	CG	10.2	1.7	9.85	1.3				
TUG (s)	XKS	9.97	0.7	8.75	0.5	0.000	10.0	0.323	Large effect
	NSS	9.18	0.6	9.00	0.6				
	CG	8.85	1.6	9.2	1.6				
Sit-and-reach (cm)	XKS	7.11	0.8	9.75	1.2	0.000	37.3	0.640	arge effect
	NSS	7.18	0.6	6.92	0.6				
	CG	8.84	1.7	8.54	1.6				
2-min step (rep)	XKS	69.8	9.1	78.9	8.1	0.000	9.85	0.319	Large effect
	NSS	70.3	8.1	73.3	8.8				
	CG	68.9	9.1	64.5	12.0				

Note: MIHS: maximal isometric handgrip strength. TUG: timed up-and-go. Rep: repetitions. ηp^2^: partial eta square. SD: standard deviation. XKS: Xbox Kinect sports. NSS: Nintendo Switch sports. CG: control group.

## Data Availability

The datasets generated during and/or analyzed during the current research are available from the corresponding author upon reasonable request.
